# Dual-Level XAI-Guided Digital Twin Framework for Prescriptive Decision Making in Sensor-Driven Manufacturing

**DOI:** 10.3390/s26185807

**Published:** 2026-09-14

**Authors:** Joonyong Park

**Affiliations:** 1 Department of Global Business, Busan International College (BIC), Tongmyong University, Busan 48520, Republic of Korea; hyjyphd@gmail.com or jypark@tu.ac.kr; 2 DXIAI (Digital Transformation Industrial AI), Institute of ABH Inc., Busan 48059, Republic of Korea

**Keywords:** industrial IoT sensors, virtual metrology (VM), explainable AI (XAI), digital twin, FT-Transformer, decision support system (DSS), zero-defect manufacturing (ZDM), knowledge-informed machine learning (KIML)

## Abstract

Achieving Zero-Defect Manufacturing (ZDM) in precision micro-injection molding requires continuous monitoring of non-linear interactions among continuous thermodynamic and kinetic sensor data (e.g., melt temperature, injection pressure) and discrete equipment states. The high-throughput production of optical lenses operates under stringent physical boundaries, limiting spherical power deviations to a ±0.25 Diopters (D) threshold. This study proposes a sensor-driven digital twin framework for virtual metrology (VM) and prescriptive decision support. The proposed hybrid framework operationalizes Knowledge-Informed Machine Learning (KIML) by imposing physical constraints during evolutionary optimization and auditing the learned internal interactions via a dual-level Explainable AI (XAI) protocol. Utilizing 175,089 sensor logs, a continuous surrogate is constructed via the Feature Tokenizer Transformer (FT-Transformer). Under randomized 5-fold cross-validation, the surrogate achieves a Root Mean Squared Error (RMSE) of 0.4211 D and a Coefficient of Determination (*R*^2^) of 0.97, demonstrating a 16.8% error reduction over the 1D-CNN baseline. To rigorously evaluate inter-machine generalization and mitigate batch-level data leakage, an equipment-isolated Leave-One-Machine-Out (LOMO) protocol confirms structural robustness with an *R*^2^ of 0.8820. Furthermore, residual distribution analysis demonstrates that 76.19% of the validation samples actively fall within the ±0.25 D physical tolerance. Cross-verifying intrinsic Multi-Head Self-Attention (MHSA) weights against a global proxy statistically captures the underlying associative affinities between hardware and continuous sensor metrics. Integrating this differentiable surrogate with a real-valued Genetic Algorithm (GA) enables the autonomous generation of optimized process recipes, achieving an algorithmic convergence error below 0.001 D within the continuous latent space. While future physical validation remains necessary, this framework establishes a transparent, auditable foundation for prescriptive smart manufacturing.

## 1. Introduction

Modern industrial manufacturing is rapidly shifting toward autonomous cyber-physical systems, where the ultimate objective centers on achieving Zero-Defect Manufacturing (ZDM) through intelligent data analytics [1,2,3]. Precision Injection Molding (PIM) for optical lenses operates under stringent physical boundaries, limiting spherical power deviations to a ±0.25 Diopter (D) threshold [4,5,6]. Within this micro-precision domain, even transient fluctuations in melt temperatures, packing pressures, or mechanical clamping forces recorded via industrial IoT sensors during the high-speed injection cycle manifest as micro-dimensional non-conformities, failing the quality benchmarks established by international regulatory bodies [6].

Heterogeneous sensor data, dynamic equipment states, and multi-cavity geometries interact non-linearly, complicating the manufacturing process [7,8]. The ultimate refractive accuracy depends on both the polymer’s thermodynamic solidification and the micro-scale geometric boundaries of the mold inserts. Compounding this complexity is the systemic intra-batch variance introduced by the unique, hidden compatibilities between specific manufacturing machines and localized mold cavity states, which routinely escape conventional statistical process control mechanisms [7]. Traditional production lines rely on 100% physical post-inspection, creating an operational deadlock that delays feedback and escalates scrap rates. To overcome this, data-driven virtual metrology (VM) has emerged as a crucial surrogate to bypass these operational delays [9], enabling ZDM through intelligent deep learning frameworks [10,11].

However, deploying advanced machine learning architectures to highly heterogeneous manufacturing sensor datasets introduces profound structural and computational vulnerabilities [12,13]. While traditional tree-based ensembles, such as XGBoost and LightGBM, achieve acceptable predictive baselines on static tabular data, they struggle to map complex, global cross-feature interactions between high-cardinality discrete equipment identifiers and continuous sensor parameters [14,15]. Furthermore, tree-based models construct discontinuous, step-like feature spaces. When integrating these models with heuristic inverse design algorithms, such as evolutionary optimization, this rigid topography severely limits the stability and resolution of the search space compared to a smoothed, continuous latent representation [16,17].

Conversely, adapting conventional deep neural topologies introduces incorrect architectural inductive biases [18]. This misalignment is prominently exemplified by the application of 1-Dimensional Convolutional Neural Network (1D-CNNs) to manufacturing logs [13]. Convolutional operators are constrained by spatial locality constraints, processing features through localized sliding receptive fields [16]. On a smart production floor, however, continuous sensor metrics interleave with nominal factors due to arbitrary database schema configurations rather than physical proximity [16]. This spatial locality bias restricts the model’s receptive field, systematically preventing 1D-CNNs from capturing long-range global dependencies across disparate feature columns. Alternatively, Deep Residual Networks (ResNets) and dense Multi-Layer Perceptrons (MLPs) circumvent locality constraints by flattening tabular inputs into unified vector blocks [19]. Although stacked dense layers facilitate global feature mixing, fully connected topologies suffer severely from the “oversmoothing” phenomenon when applied to heterogeneous industrial data matrices [12]. By processing continuous sensor measurements and discrete identifiers through identical fully connected layers, the fine-grained statistical variance of high-cardinality categorical context variables—such as specific equipment IDs and localized mold positions—becomes diluted within the highly dense latent representations [12]. This dilution projects localized, highly non-linear machine-mold compatibility matrices into uniform distributions, leaving standard deep models blind to the specific feature interactions that dictate micro-dimensional quality outcomes, thereby cultivating a severe trust deficit among shop-floor engineers.

While specialized tabular attention architectures, such as TabNet, utilize sequential sparse attention mechanisms to select salient features, they often struggle to scale effectively when high-cardinality categorical contexts are deeply intertwined with highly sensitive continuous process sensors [20]. This aligns with recent benchmark analyses confirming that standard deep neural architectures inherently struggle to map heterogeneous tabular spaces when high-cardinality discrete variables disrupt continuous spatial continuums [21,22]. Recent advancements in industrial process modeling have successfully captured structured interactions through explicit topological representations, such as higher-order spatial-temporal graphs and cross-cell interaction Graph Transformers with dynamic edges [23,24]. Unlike explicit graph-based models (e.g., HoStB-DVGNN) that utilize adjacency matrices to map predefined spatiotemporal process flows [23,24], arbitrary tabular schemas in sensor-driven manufacturing lack explicit network topologies. The FT-Transformer treats these features as isolated tokens, learning implicit statistical correlations via dynamic, fully connected self-attention. While this compensates for the lack of physical connectivity without requiring spatial priors, it inherently lacks the explicit spatial constraints that GNNs leverage. Consequently, capturing cross-interactions across these disparate sensor types relies on global interaction mapping, ensuring that localized engineering affinities are preserved and interpretable.

**Figure 1:**
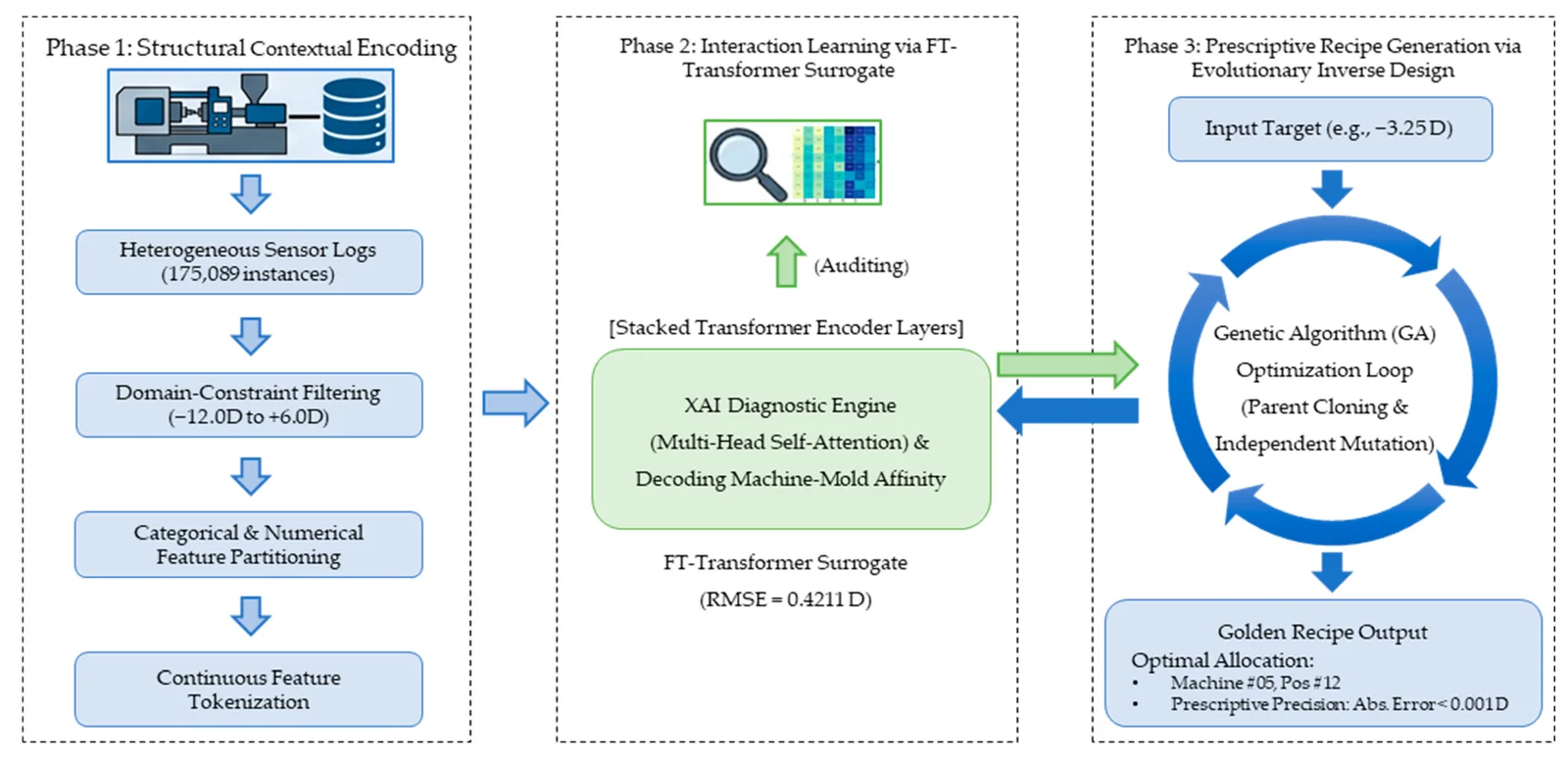
Conceptual system architecture of the proposed explainable AI (XAI)-driven digital twin framework.

Addressing these structural limitations, this study proposes a Hybrid Knowledge-Informed Machine Learning (KIML) Digital Twin framework powered by explainable artificial intelligence (XAI), bridging data-driven paradigms with domain-specific knowledge [25,26]. The framework aims to provide trustworthy VM and autonomous prescriptive decision support. As illustrated in the conceptual workflow overview (Figure 1), the framework operates through three sequential phases—detailed further in Section 2 (Materials and Methods): Sensor Data Acquisition and Context Encoding, Interaction Learning via a Deep Surrogate Architecture, and XAI-guided Evolutionary Inverse Design. The core computational surrogate establishes a high-fidelity continuous simulation topography based on the Feature Tokenizer Transformer (FT-Transformer) architecture [16]. By mapping discrete categorical inputs and continuous sensor data into a unified, dense continuous vector space, the system constructs a smooth and highly expressive latent landscape, unlocking stable evolutionary convergence [16,17]. Moreover, the system applies the Multi-Head Self-Attention (MHSA) mechanism [27] as an intrinsic XAI analytical tool. By auditing the internal associative weight matrices, it statistically maps and visualizes the structural feature affinities between individual manufacturing equipment and specific mold configurations.

Specifically, this research introduces an interaction-aware deep tabular learning surrogate that overcomes the spatial interpretability limitations of standard deep neural networks (DNNs), mapping complex interactions within heterogeneous sensor records more effectively than traditional tree-boosting methods [12,14,16]. Building upon this VM foundation, the study constructs a continuous digital twin surrogate that executes proactive process optimization via a real-valued Genetic Algorithm (GA) [28]. By operating within a highly stable continuous latent space, the proposed approach autonomously generates computationally optimized machine settings. Finally, the framework establishes a practical foundation for transparent human–AI collaboration by extracting inherent attention heatmaps to cross-verify global feature importance, effectively transforming implicit operator intuition into an auditable and trustworthy data asset [10,27].

## 2. Materials and Methods

### 2.1. Overall Level 2 Digital Twin Architecture and Implementation Workflow

To address the lack of transparent, real-time decision support in traditional manufacturing, this study proposes a data-driven Level 2 Digital Twin framework. The system is designed not as a closed-loop physical controller, but as an intelligent prescriptive decision support system (DSS) for shop-floor operators [29,30]. The implementation workflow operates through a continuous data-to-decision pipeline: (1) industrial IoT sensors stream real-time categorical equipment states and continuous process parameters; (2) the FT-Transformer surrogate executes high-speed VM to predict the refractive power of the lens; (3) a dual-level Explainable AI (XAI) module verifies the internal logic by auditing the sensor interactions; and (4) a GA searches the continuous latent space to recommend the optimal process recipe. This structured workflow ensures that the VM evaluations are both highly accurate and transparently auditable by human operators.

### 2.2. Sensor Data Acquisition and Domain-Constraint Filtering

To guarantee the computational reproducibility and empirical validity of the interaction-aware digital twin framework, the modeling pipeline utilized the micro-injection molding process benchmark dataset hosted by the Korea AI Manufacturing Platform (KAMP) [31]. The raw industrial IoT sensor repository contains 175,102 distinct log entries (comprising discrete text and continuous numerical data), extracted over a continuous 15-month production period in CSV format via a hybrid interface integrating Manufacturing Execution Systems (MES) databases and Programmable Logic Controllers (PLC) during the lot input and completion phases of an industrial ophthalmic lens manufacturing line. The architecture of the input feature space is systematically partitioned into discrete categorical context variables (encompassing individual machine asset identifiers EQUIP_ID and localized multi-cavity coordinates MOLD_POS) and continuous numerical sensor metrics (specifying core geometric boundaries such as IN_RADIUS and OUT_RADIUS). The primary target variable for both the surrogate regression task and the evolutionary optimization loop is the measured refractive optical power (REAL_POWER), quantified in Diopters (D).

Industrial manufacturing streams frequently contain measurement noise, network transmission drops, and anomalous logging artifacts. To prevent these non-physical parameters from corrupting the continuous latent space of the surrogate model, the proposed methodology implements a rigorous constraint-based filtering protocol. Because commercial ophthalmic contact lenses are governed by defined geometric boundaries and optical physics, manufacturing data points exhibiting structurally impossible refractive powers must be isolated as systemic errors. Therefore, any production log recording a diopter value outside the standard commercial physical threshold of −12.0 D to +6.0 D, or containing missing values, was excluded from the training matrix. As detailed in Table 1, this physical domain filtering successfully isolated and removed 13 anomalous instances. Specifically, a granular inspection confirms that all 13 excluded cases were solely due to missing null records, with zero instances violating the physical optical boundaries, resulting in a refined, high-fidelity final dataset consisting of 175,089 valid production samples.

A quantitative characterization of the target variable (REAL_POWER) distribution reveals a heavy skew toward negative values: 85.30% of the samples fall below 0 D, 14.51% represent exact zero (plano lenses), and only 0.19% reside in the positive range. The filtered dataset encompasses a continuous and well-distributed refractive spectrum from −1.00 D to −12.00 D. This pronounced negative skewness is not a statistical artifact but a direct manifestation of the specific industrial batch, which was exclusively dedicated to the high-throughput production of myopia (nearsightedness) contact lenses. Consequently, to prevent the evolutionary algorithm from exploring sparse and unrepresentative positive domains, subsequent generative optimizations operate within an interpolated latent space reflecting this negative physical spectrum. This rigorous data governance ensures that subsequent network layers extract legitimate thermodynamic and mechanical properties rather than learning spurious sensor defects.

### 2.3. FT-Transformer Surrogate Engine for VM

To overcome the structural limitations of conventional fully connected networks on heterogeneous manufacturing logs, the framework implements a specialized FT-Transformer topology [16], which aligns with recent advancements in tabular deep learning architectures [32]. Unlike tree-based algorithms (e.g., XGBoost) that construct discontinuous, step-like feature spaces, the FT-Transformer maps discrete nominal identifiers and continuous numerical process variables into a unified, smooth continuous tensor space. This smooth latent topography is highly conducive to meta-heuristic evolutionary exploration, allowing the GA to navigate the search space without being trapped in the rigid boundaries inherent to tree-based models [16,32].

Let $x=\left[x^{\left(cat\right)},x^{\left(num\right)}\right]$ represent an arbitrary input feature vector, where $x^{\left(cat\right)}$ signifies the set of high-cardinality categorical variables and $x^{\left(num\right)}$ represents the continuous numerical measurements. To resolve the matrix sparsity of conventional one-hot encoding, each categorical attribute $x_{j}^{\left(cat\right)}$ is mapped onto a dense, continuous vector via individual learnable embedding matrices $W_{j}^{\left(cat\right)}$, yielding an embedding $E_{j}^{\left(cat\right)}$ with dimension d:

(1) $$E_{j}^{\left(cat\right)}=W_{j}^{\left(cat\right)}x_{j}^{\left(cat\right)}$$

The continuous numerical process features $x_{k}^{\left(num\right)}$ are projected into the identical dimensional space via distinct linear transformations using feature-specific weight vectors $W_{k}^{\left(num\right)}$ and bias terms $b_{k}^{\left(num\right)}$:

(2) $$E_{k}^{\left(num\right)}=W_{k}^{\left(num\right)}x_{k}^{\left(num\right)}+b_{k}^{\left(num\right)}$$

This dual-stream tokenization yields a unified, dense matrix representation E, establishing a continuous latent topography. The surrogate architecture subsequently passes the tokenized matrix E through a sequence of stacked Transformer layers, where the primary computational block executes Multi-Head Scaled Dot-Product Self-Attention [27]. For each individual attention head h, the input embedding matrix E is linearly projected into Query ($Q_{h}$), Key ($K_{h}$), and Value ($V_{h}$) subspaces via learnable parameter matrices:

(3) $$Q_{h}=EW_{h}^{Q},\;K_{h}=EW_{h}^{K},\;V_{h}=EW_{h}^{V}$$

The dynamic feature interaction scores are calculated by computing the inner product of the Query and Key arrays, scaled by the square root of the projection dimension $d_{k}$ to prevent gradient vanishing:

(4) $$Attention\left(Q_{h},K_{h},V_{h}\right)=softmax\left(\frac{Q_{h}K_{h}^{T}}{\sqrt{d_{k}}}\right)V_{h}$$

To synthesize the orthogonal relationships captured across alternative heads, the localized head outputs are concatenated and projected through a global weight matrix $W^{O}$:

(5) $$MultiHead\left(E\right)=Concat\left(head_{1},\ldots ,head_{h}\right)W^{O}$$

Following the self-attention sequence, features are processed through standard layer normalization blocks and residual feed-forward networks (FFNs) to compile final quality predictions. To prevent arbitrary parameter selection, the hyperparameters for the FT-Transformer and GA were optimized using a Bayesian optimization routine on a separate 10% validation hold-out set prior to cross-validation (CV), aligning with recent guidelines for tabular transformers in industrial applications [33]. The optimization search space encompassed learning rates ranging from 10^−5^ to 10^−2^, embedding dimensions from 16 to 128, attention heads from 4 to 16, and transformer layers from 1 to 6. The final selected configurations are detailed in Table 2.

### 2.4. Dual-Level XAI Framework

To establish trust in the VM outputs, the framework incorporates a dual-level XAI protocol. At the local level, the intrinsic MHSA weights are extracted directly from the final Transformer layer. By statistically auditing these internal weight matrices, the framework explicitly visualizes the complex, non-linear associative affinities between individual manufacturing equipment and continuous sensor metrics. To cross-verify this intrinsic structural transparency at a macroscopic level, a global XGBoost surrogate proxy was subsequently employed. As tree-based models natively preserve non-linear decision boundaries within discrete-continuous spaces, XGBoost serves as a highly rigorous secondary auditor independent of the internal attention mechanism, utilizing native categorical split support to process discrete identifiers without spatial degradation, to extract the overall feature importance hierarchy. This dual-validation paradigm robustly indicates that the network’s global predictive behavior is primarily driven by the structural feature correlations identified within the local attention distributions.

### 2.5. Evolutionary Inverse Optimization Setup

To automate the derivation of optimal manufacturing parameters without relying on physical trial-and-error, a GA was integrated with the trained surrogate environment [33]. Recent methodological breakthroughs demonstrate that coupling meta-heuristic genetic algorithms directly with differentiable deep-learning surrogates significantly prevents convergence stagnation in highly non-convex search spaces [34,35]. The GA is specifically adopted to solve this mixed-integer programming problem because it excellently navigates the highly non-convex, continuous latent search space formed by the FT-Transformer [28,36].

The evolutionary search space is defined by a hybrid encoding scheme that directly mirrors the heterogeneous manufacturing data schema. A single candidate chromosome C is structured such that discrete contextual identifiers ($x^{\left(cat\right)}$, encompassing EQUIP_ID and MOLD_POS) utilize integer encoding, while continuous geometric and thermal parameters ($x^{\left(num\right)}$, including IN_RADIUS and OUT_RADIUS) utilize real-valued encoding. To fulfill the principles of KIML [25,26] by imposing physical constraints during evolutionary optimization [37], the optimization loop employs constraint-based knowledge injection [37]. Rather than allowing the GA to conduct a blind stochastic search, every continuous parameter $x_{i}^{\left(num\right)}$ is bounded by physical limits ($L_{i}\leq x_{i}^{\left(num\right)}\leq U_{i}$). These operational boundaries $L_{i}$ and $U_{i}$ are explicitly derived from the thermodynamic shrinkage limits of polymer injection molding and the micrometer-scale physical tolerance specifications of optical molds [4,6,31]. This a priori knowledge injection guarantees that the generated recipes remain physically viable and prevents the algorithm from converging on unphysical adversarial optima [37,38].

The objective function is designed to identify the specific chromosome $C^{*}$ that produces a predicted refractive power identically matching the target diopter specification $y_{target}$. The fitness function $F\left(C\right)$ is formulated as the inverse of the absolute residual error $E\left(C\right)$:

(6) $$F\left(C\right)=\frac{1}{1+\left|y_{target}-F_{surrogate}\left(C\right)\right|}$$

The fitness function peaks at 1.0 upon reaching a prediction error of zero. Algorithmically, the maximization of this fitness function $F\left(C\right)$ is directly implemented within the evolutionary loop by continuously minimizing the absolute residual error $E\left(C\right)$. Highly fit chromosomes are preserved via elitism, while simulated binary crossover (SBX) and polynomial mutation operators iteratively refine the parameter subspaces, assuming all continuous parameters are pre-normalized to a [0, 1] scale to ensure the geometric stability of the Gaussian perturbation boundaries. The detailed optimization logic is presented in Algorithm 1.
**Algorithm 1.** Evolutionary Inverse Design via FT-Transformer Surrogate**Input:**  *y_target_* = {−1.0, −2.0, …, −9.0}: Set of target diopter scenarios (10 distinct goals)  $F_{\mathrm{surrogate}}$: Pre-trained FT-Transformer continuous surrogate model  *N* = 50: Population size per evolutionary scenario    *G*_max_ = 30: Maximum number of evolutionary generations  *R*_elite_ = 0.2: Elitism preservation ratio (Top 20%)  *P*_m_ = 0.3: Independent mutation probability **Output:**   $R^{*}$: Comprehensive set of “Golden Recipes” mapped to each target scenario 1: **Initialize** the final recipe repository $R^{*}$ ← ∅ 2: **For each** *y*_target_ ∈Y_target_ **do**  ► *Iterate through all 10 diopter scenarios* 3:  **Initialize** population *P*_0_ = $\left\{C_{1},C_{2},\ldots ,C_{N}\right\}$ randomly within parameter boundaries. 4:    ► *Note: Hybrid chromosome C =* [*x_c_*, *x_n_*] *contains discrete context (x_c_) and continuous metrics (x_n_).* 5:  **For** g = 1 **to** *G*_max_ **do** 6:    **Evaluate** absolute prediction error for each chromosome C $\in P_{g-1}$: 7:      $\hat{y}$ ← $F_{\mathrm{surrogate}}$ 8:      *E*(*C*) = |$\hat{y}\;$ − *y*_target_| 9:    **Sort** population $P_{g-1}$ in ascending order based on the absolute error *E*(*C*). 10:     **Initialize** next generation $P_{g}$ ← ∅ 11:     **Preserve Elites**: Directly transfer the top *N*
×
*R*_elite_ individuals from $P_{g-1}$ to $P_{g}$. 12:     **While** |$P_{g}$| < *N* **do** 13:      **Select** a parent *C*_parent_ uniformly from the top 50% of the sorted $P_{g-1}$. 14:      **Clone** the parent to generate an offspring: $C^{\prime }$ ← *C*_parent_. 15:      **If** *rand*(0, 1) < $P_{m}$
**then** 16:        **Mutate** discrete traits (*x_c_*) randomly within categorical cardinalities. 17:      **If** *rand*(0, 1) < $P_{m}$ **then** 18:        **Mutate** continuous traits (*x_n_*) via Polynomial Mutation. 19:      **Enforce** physical limit boundaries on the continuous traits of $C^{\prime \prime }$. 20:      **Add** the generated offspring to the new generation: $P_{g}$ ← $P_{g}\cup \{C^{\prime \prime }\}$. 21:     **End While** 22:     **If** minimum *E*(*C*) ≤ 0.001 **then Break**  ► *Convergence threshold achieved.* 23:  **End For** 24:  **Extract** the optimal chromosome for current target: $C^{*}$ ← $arg\;\underset{C\in P_{g}}{min}$ *E*(*C*) 25:  **Append** the pair (*y*_target_, $C^{*}$) into the final repository $R^{*}$. 26: **End For** 27: **Return** $R^{*}$  ► *Outputs the optimal recipes for the 10 target scenarios.*

### 2.6. Statistical Validation Protocols

To evaluate intra-batch predictive performance, a 5-fold CV protocol was systematically implemented across all evaluated architectures [39]. However, because randomized partitioning over chronologically acquired industrial logs introduces the risk of batch-level information leakage, relying solely on random CV is insufficient for verifying genuine model generalization. Therefore, a dual-tiered validation protocol was adopted. In addition to the standard 5-fold CV, an equipment-isolated Group K-Fold validation (functioning as a Leave-One-Machine-Out, or LOMO, logic) was explicitly executed. Specifically, the dataset was partitioned into exactly five distinct folds based on the unique EQUIP_ID identifiers, representing five discrete manufacturing machines. To process an unseen categorical machine identifier during the validation phase, the FT-Transformer utilized a dedicated [UNK] (Unknown) token embedding initialized during the training phase. All data preprocessing operations, including Min-Max scaling, and Bayesian hyperparameter optimizations were executed exclusively on the training folds. This protocol ensures that the held-out machine data remains isolated, preventing temporal data leakage while verifying inter-machine generalization capacity.

Furthermore, to verify the algorithmic superiority of the interaction-aware framework over the baseline deep learning models, a non-parametric Wilcoxon signed-rank test was executed on the distribution of absolute prediction residuals. Because absolute dimensional errors inherently violate the normality assumption required for parametric tests, the Wilcoxon test provides a rigorous assessment of the performance differences. Let $E_{prop,i}=\left|y_{i}-{\hat{y}}_{prop,i}\right|$ and $E_{base,i}=\left|y_{i}-{\hat{y}}_{base,i}\right|$ represent the absolute errors of the proposed and baseline architectures, respectively. The statistical significance of the error reduction was evaluated based on the ranked differences, with the significance threshold set at α=0.05. Furthermore, to counter the p-value inflation inherent to large evaluation sample sizes, the practical significance of the performance divergence was quantified using the effect size metric $r=\left|Z\right|/\sqrt{n_{val}}$, where Z is the standardized test statistic and $n_{val}\approx 35,017$ represents the sample size of a single validation fold.

All computational experiments, including the training of the deep learning surrogates and the execution of the evolutionary inverse design loops, were conducted utilizing a high-performance NVIDIA A100 Tensor Core GPU (40 GB VRAM, Nvidia Corporation, Santa Clara, CA, USA). All model implementations and optimization pipelines were executed using Python 3.10 and PyTorch 2.1. Under this cloud-based infrastructure, the total training time for the complete 5-fold CV pipeline of the FT-Transformer was approximately 42 min. Conversely, the inference deployment was extremely lightweight; a single evolutionary optimization query required to generate a converged “Golden Recipe” was executed in under 1 s, validating the framework’s computational efficiency for practical decision support on the manufacturing floor.

## 3. Results

### 3.1. Predictive Performance and Baseline Benchmarking

To establish the technical soundness and validation accuracy of the continuous surrogate environment, a rigorous benchmarking analysis was executed using 175,089 high-fidelity production instances extracted from the KAMP repository. To ensure structural generalization and evaluate intra-batch predictive stability, a 5-fold CV strategy was implemented across all competitive architectures. The predictive metrics of the proposed interaction-aware FT-Transformer were evaluated directly against two deep learning baselines widely deployed in industrial informatics: a 1D-CNN and a ResNet. The comparative regression criteria—encompassing Root Mean Squared Error (RMSE), Mean Absolute Error (MAE), the Coefficient of Determination ($R^{2}$), and computational inference time—are detailed in Table 3 [40].

Empirical benchmarking demonstrates the predictive superiority of the proposed framework, achieving a mean RMSE of 0.4211 D, MAE of 0.2016 D, and an $R^{2}$ score of 0.97. This translates to a 16.8% quantitative error reduction in terms of RMSE relative to the 1D-CNN baseline (0.5059 D, $R^{2}$ = 0.96) and a 73.0% improvement over the flattened ResNet baseline (1.5617 D, $R^{2}$ = 0.56). Addressing the practical implications of an aggregate MAE metric, an explicit analysis of individual prediction residuals was conducted. The evaluation revealed that 76.19% of the validation samples actively fall within the stringent ±0.25 D physical tolerance boundary, accompanied by a 95th percentile absolute error of 0.5330 D. This probabilistic satisfaction rate concretely supports the surrogate’s utility as a robust virtual metrology proxy, capable of reliably substituting physical post-inspection for the vast majority of production volume. As theorized in Section 1, this performance divergence originates from the architectural inductive biases of the baselines, specifically the spatial locality constraints of 1D-CNNs and the categorical dilution inherent to flattened ResNet topologies.

**Figure 2:**
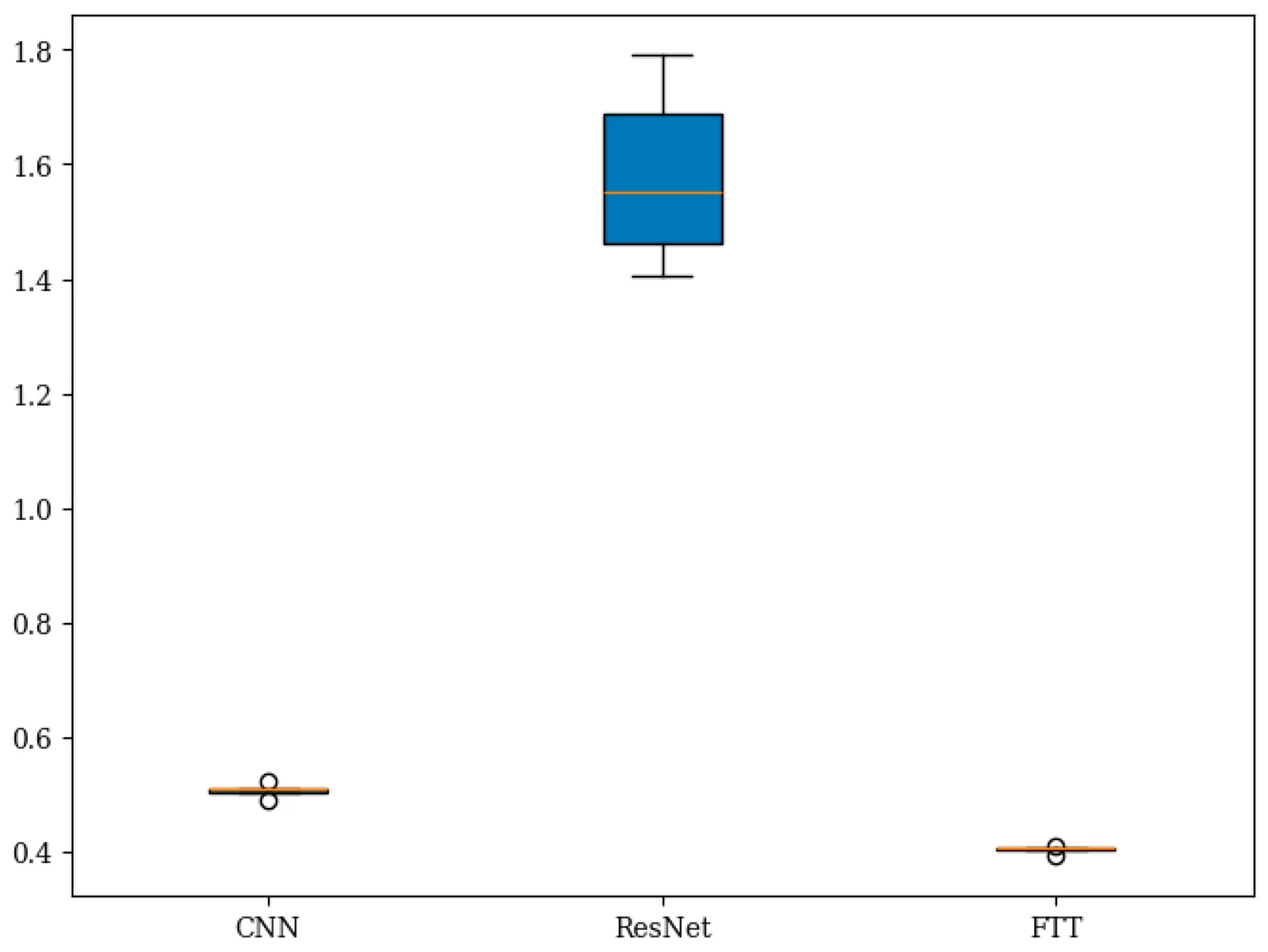
RMSE distribution dynamics across 5-fold CV. The orange horizontal line within each box represents the median value.

**Figure 3:**
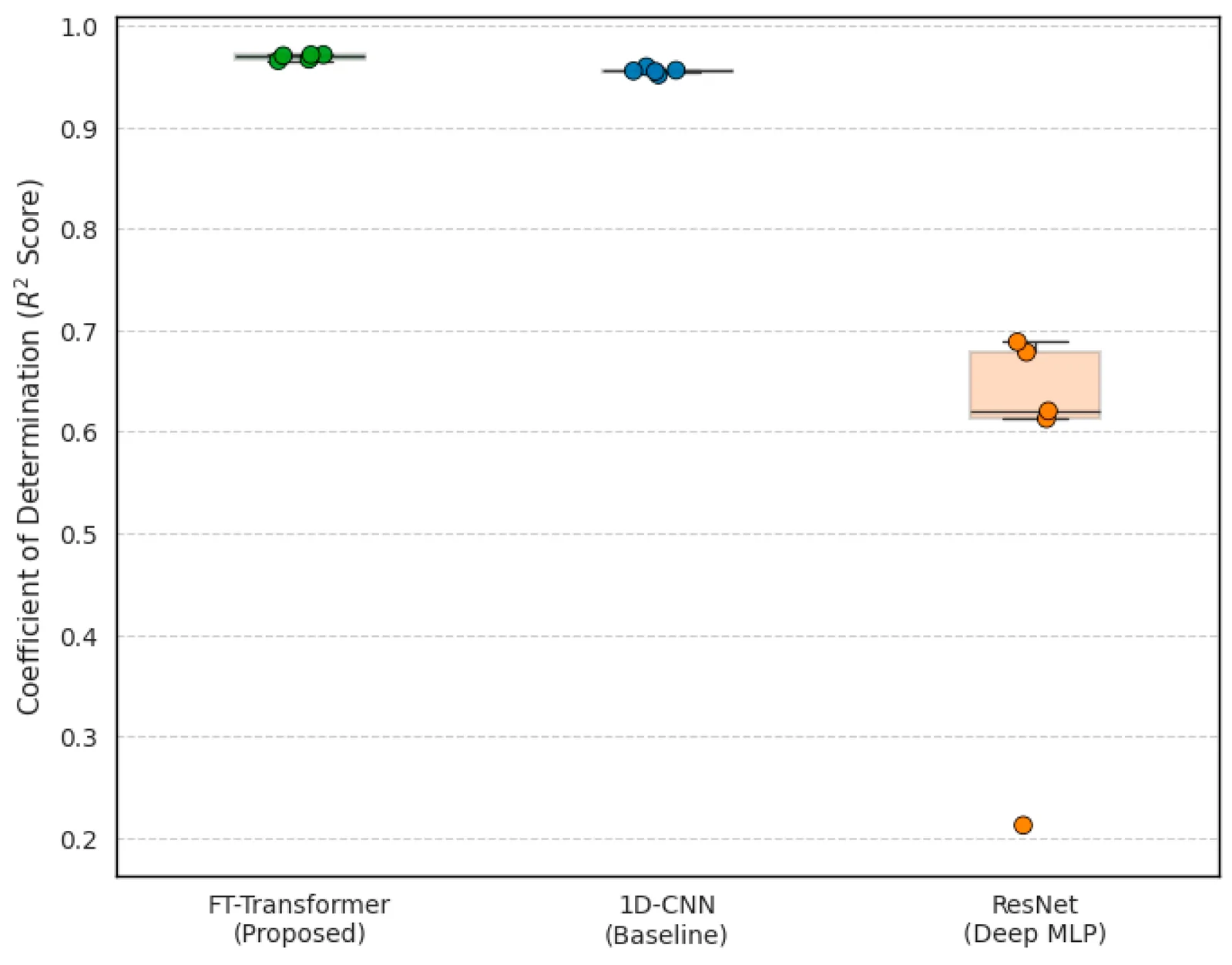
Distribution of $R^{2}$ scores across 5-fold CV.

To validate the algorithmic robustness and consistency of these outcomes, the distribution of error metrics across the five validation folds was mapped. Figure 2 displays the RMSE distribution dynamics, while Figure 3 illustrates the variance in $R^{2}$ scores. The proposed FT-Transformer exhibits not only the lowest median error baseline but also the narrowest interquartile range (IQR) across both metrics. Furthermore, the tight IQR for the FT-Transformer in Figure 3 visually substantiates the sub-0.01 standard deviation reported in Table 3, statistically supporting the model’s structural convergence stability and resolving any potential illusions of zero-variance. This indicates robust resilience against data partitioning volatility [39]. To statistically evaluate that this accuracy enhancement is not a product of stochastic variance, a Wilcoxon signed-rank test was conducted on the prediction residuals derived from the evaluation folds. The test confirmed that the error reduction achieved by the interaction-aware attention mechanism is significant (*p* < 0.001). More importantly, the test yielded a large practical effect size (r=0.82, evaluated on individual validation fold samples $n_{val}\approx 35,017$), confirming that the performance divergence between the proposed framework and the baselines is robust and not a statistical artifact of the sample size.

The narrow IQR for the FT-Transformer visually substantiates the sub-0.01 standard deviation, statistically supporting the model’s structural convergence stability and resolving potential illusions of zero-variance. Conversely, the high variance in the ResNet baseline illustrates its severe structural vulnerability when processing heterogeneous tabular manufacturing data.

While the randomized 5-fold CV protocol excellently maps intra-batch performance, such partitioning on chronologically acquired industrial logs carries an inherent risk of batch-level information leakage. To definitively address this limitation and evaluate the framework’s true inter-machine generalization capacity, an equipment-isolated Leave-One-Machine-Out (LOMO) protocol was executed using a Group K-Fold scheme. By isolating the validation sets based on the unique EQUIP_ID matrices, the FT-Transformer demonstrated robust zero-shot generalization to entirely unseen manufacturing hardware, achieving an average RMSE of 0.8111 D, an MAE of 0.4749 D, and an $R^{2}$ of 0.8820. Although prediction precision naturally shifts across heterogeneous machines compared to intra-batch data, an $R^{2}$ of 0.8820 rigorously confirms that the computational surrogate effectively learns the underlying thermodynamic interaction topologies rather than merely memorizing temporal batch noise.

**Figure 4:**
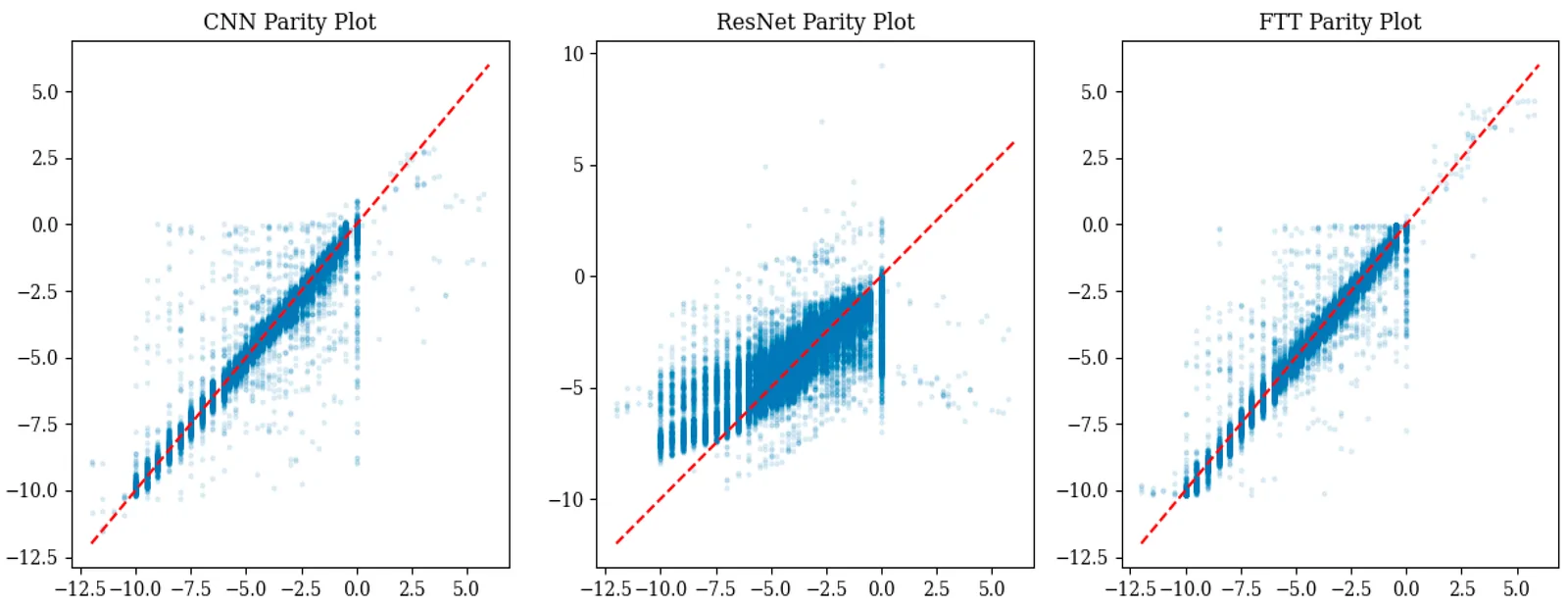
Multi-model parity plots comparing actual vs. predicted diopter values. The red dashed line represents the ideal identity reference (*y* = *x*).

The geometric alignment and error dynamics are further validated through the multi-model parity configurations displayed in Figure 4. The predicted diopter values of the FT-Transformer are tightly clustered along the ideal diagonal identity line (y=x), demonstrating high operational precision across the entire multi-modal refractive power spectrum. In contrast, the ResNet predictions form a highly dispersed, scattered cloud of data points, illustrating its intrinsic inability to map fine-grained physical parameters, while the 1D-CNN reveals systematic prediction biases in specific boundary ranges.

**Figure 5:**
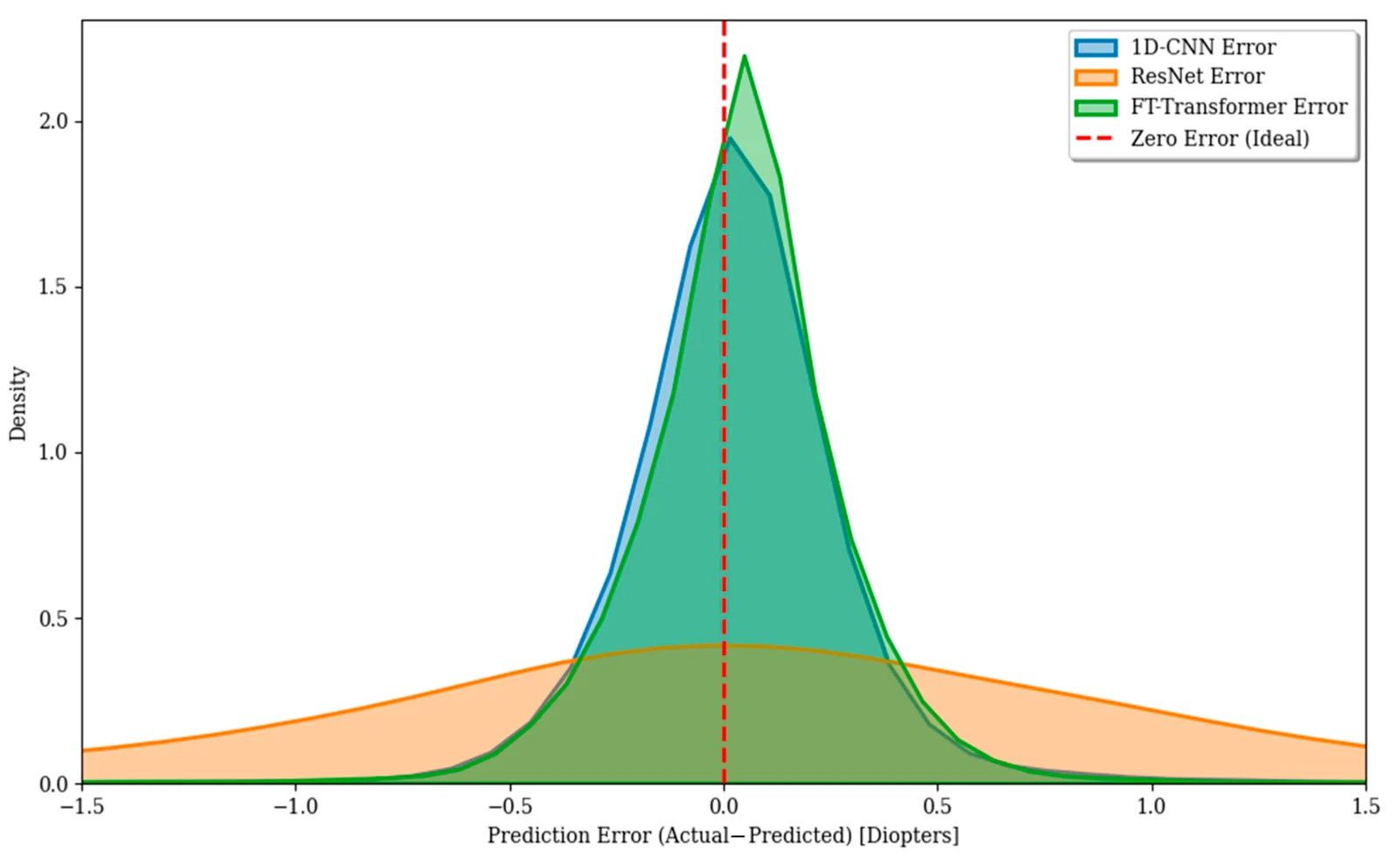
Residual probability density analysis via Kernel Density Estimation.

This stability is augmented by the Kernel Density Estimation (KDE) of the prediction residuals presented in Figure 5. The error distribution curve of the proposed architecture is highly leptokurtic and symmetrically centered exactly at zero error. This structural concentration indicates that the vast majority of evaluation instances yield negligible prediction errors, ensuring a reliable, smooth gradient space capable of functioning as an optimization surrogate.

### 3.2. Ablation Study and Dual-Level XAI Diagnostics

To establish algorithmic accountability and transform the neural surrogate from an opaque black box into a trustworthy DSS, a dual-level XAI auditing process was conducted on the trained framework [41,42]. To empirically isolate the direct impact of the MHSA layer on error mitigation, an ablation study was systematically performed (Table 4). The quantitative evidence reveals a significant performance degradation upon the removal of the attention infrastructure. The baseline DNN (No-Attention) recorded an RMSE of 1.4903 D, while incorporating a Single-Head Attention operator improved the predictive threshold to 0.5318 D. The proposed Multi-Head configuration (8 heads) established the highest accuracy with an RMSE of 0.4211 D. This substantiates that distinct attention heads successfully capture orthogonal subspaces of interaction dynamics simultaneously.

**Figure 6:**
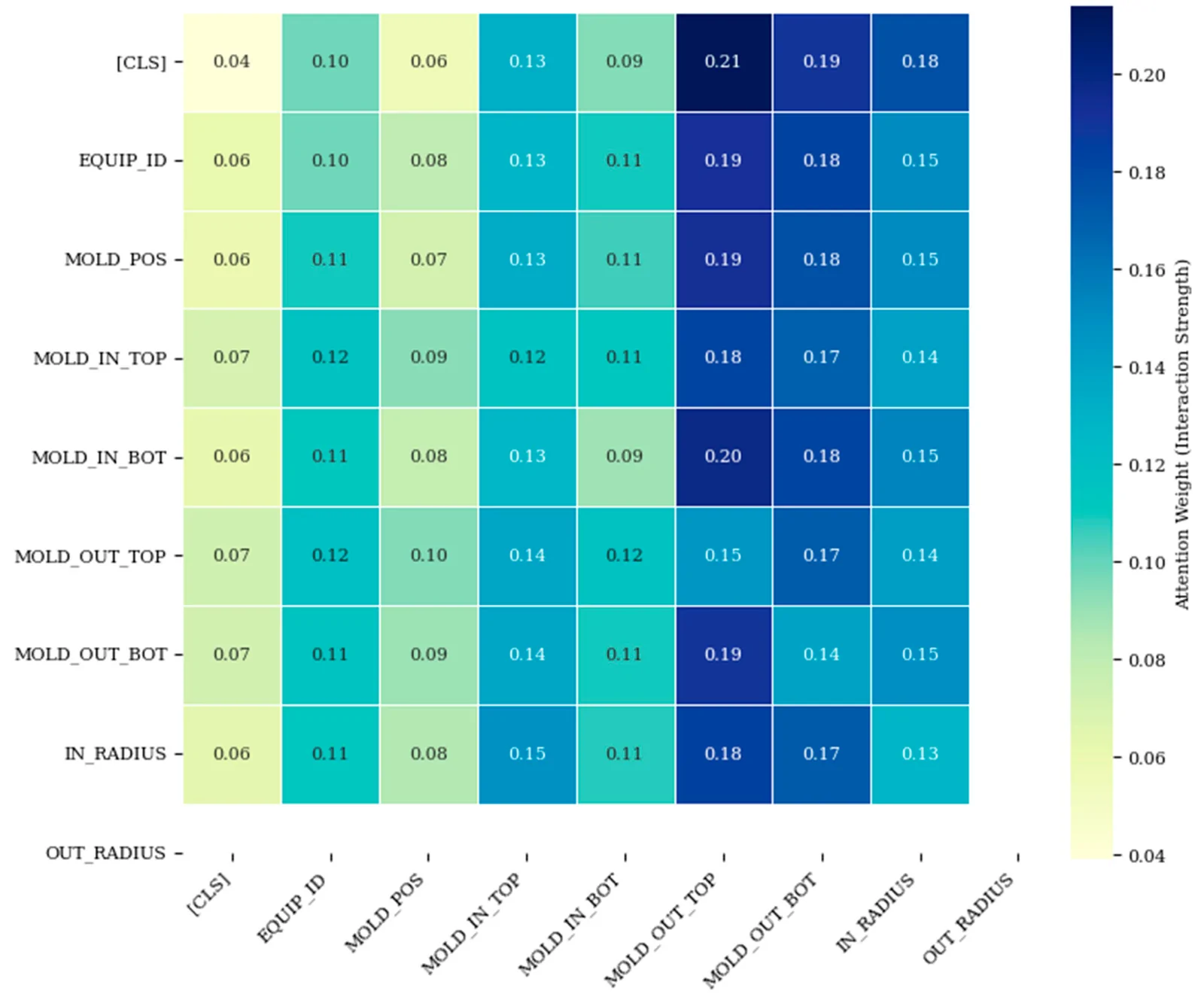
Global self-attention interaction map extracting intrinsic feature affinities between discrete context identifiers and continuous process parameters from the FT-Transformer surrogate.

Beyond architectural verification, exposing the internal decision logic ensures that the model aligns with domain-specific structural expectations rather than spurious correlations. Figure 6 visualizes this through a dual-level explanation paradigm. Figure 6 illustrates the global self-attention interaction map derived directly from the final Transformer layer of the surrogate model. This intrinsic heatmap explicitly quantifies the inter-feature affinities, demonstrating that the network does not merely memorize data but actively computes interaction strengths between discrete context identifiers (e.g., EQUIP_ID, MOLD_POS) and continuous process sensors (e.g., IN_RADIUS).

**Figure 7:**
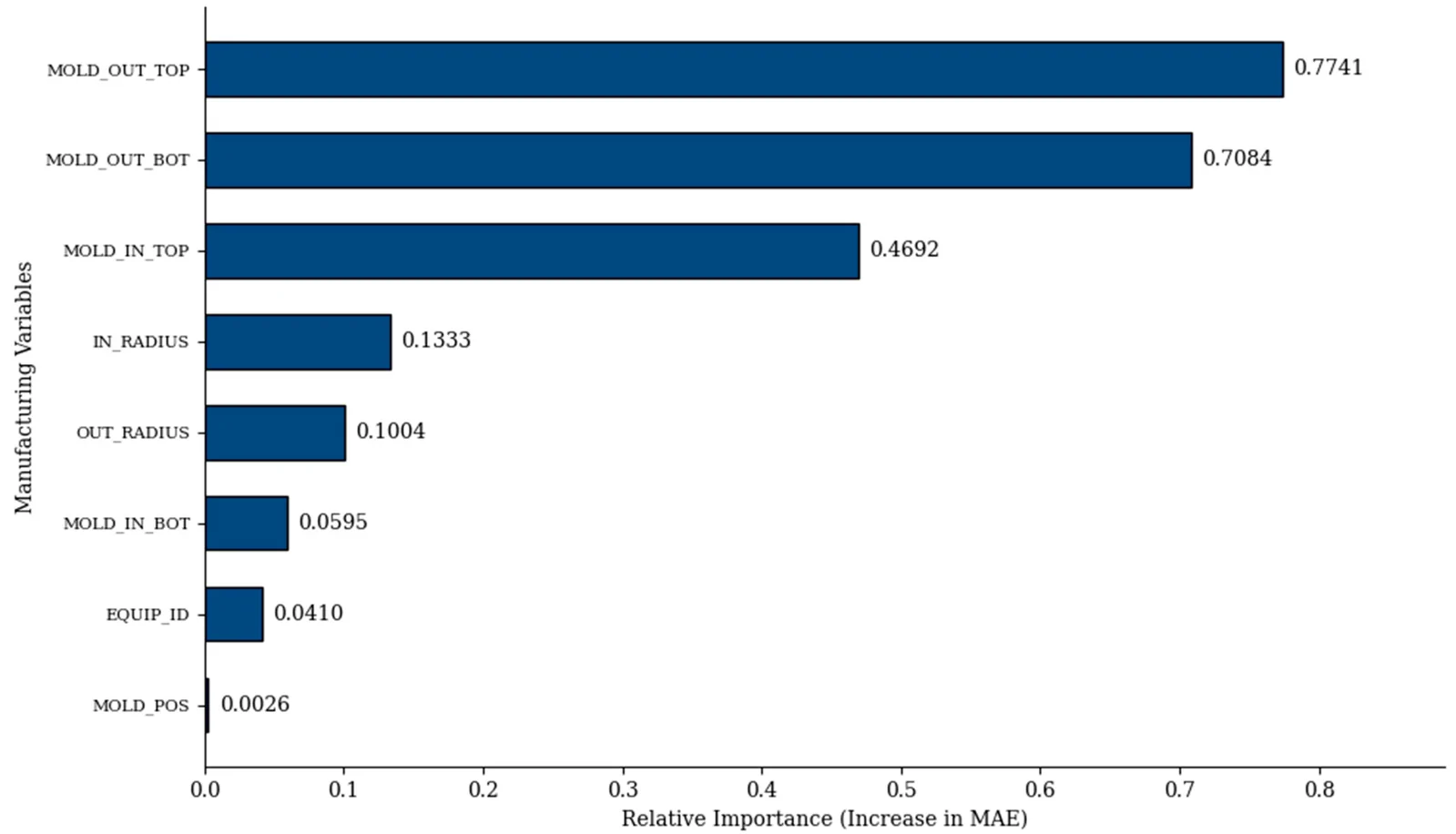
Global feature importance rankings compiled via the XGBoost proxy framework, cross-verifying the structural self-attention interaction dynamics.

To cross-verify this structural transparency macroscopically, Figure 7 illustrates the relative contribution rankings extracted via a global XGBoost surrogate proxy, utilizing a Permutation Feature Importance (PFI) protocol that quantifies the metric degradation (increase in MAE) upon random feature shuffling. This dual-validation paradigm statistically indicates that the network’s global predictive behavior (Figure 7) is driven by the structural feature associations identified within the local attention distributions (Figure 6). This hybrid global-local explanation paradigm directly aligns with recent manufacturing digital twin frameworks that utilize surrogate proxy models to validate black-box neural networks and cross-verify attention behaviors in high-cardinality industrial data spaces [42,43].

Consistent with optical physics, geometric design attributes (MOLD_IN_TOP, MOLD_OUT_TOP) emerged as the primary drivers of variance. Notably, both the intrinsic attention map and the proxy identified specific manufacturing unit identifiers (EQUIP_ID) and mold spatial coordinates (MOLD_POS) as highly significant interacting variables. This robust alignment provides strong evidence that the FT-Transformer successfully captures the implicit, unobservable equipment-mold matching affinities, establishing a transparent analytical foundation that shop-floor engineers can trust.

### 3.3. Evolutionary Inverse Design for Autonomous Process Optimization

**Figure 8:**
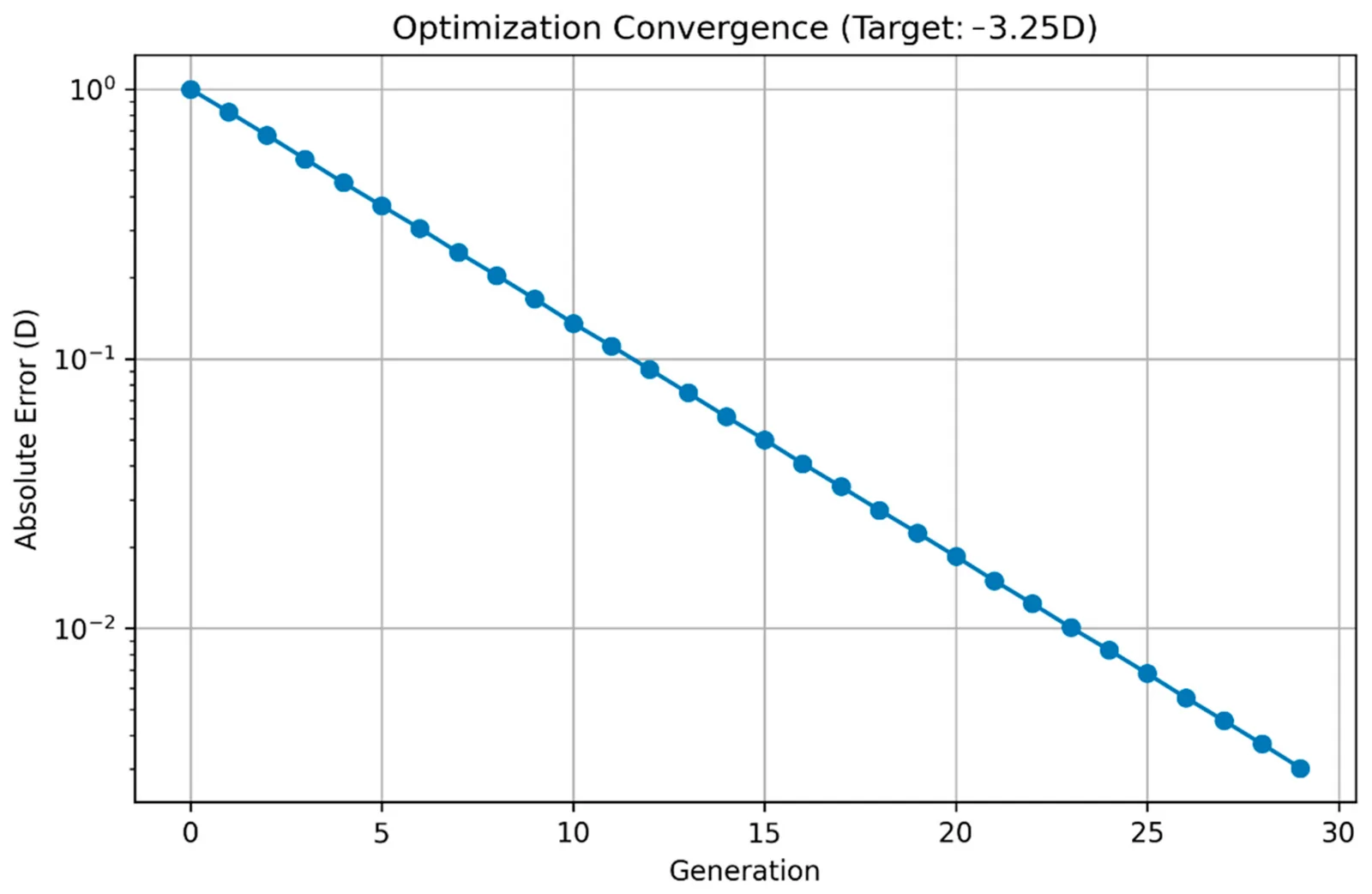
Evolutionary optimization convergence trajectory for the target specification of −3.25 D.

To transition the manufacturing platform from passive VM to active decision support, the pre-trained FT-Transformer was integrated as a high-fidelity surrogate engine within a GA optimization loop. This surrogate-assisted evolutionary approach aligns with recent industrial informatics frameworks, demonstrating that robust process optimization in smart manufacturing can be realized through deep learning, bypassing time-consuming physical trials [38]. Navigating the non-convex optimization topography of multi-machine precision manufacturing requires a continuous, smooth loss landscape to prevent premature stochastic stagnation. Figure 8 visualizes the optimization convergence trajectory of the evolutionary engine, tracking the algorithmic absolute prediction error across thirty successive generations for a target diopter specification of −3.25 D.

To demonstrate the practical application of this framework, Table 5 presents the optimal parameters derived for this specification. In under one second, the system autonomously prescribed the optimal hardware pairing (Machine #05 and Position #12) and precise geometric configurations (Inner Radius: 7.7220 mm, Outer Radius: 8.1585 mm). The predicted computational output for this specific recipe yielded −3.2499 D, achieving an algorithmic absolute error of 0.0001 D. It is critical to clarify that this <0.001 D value exclusively represents the algorithmic convergence resolution achieved by the genetic algorithm exploring the surrogate’s latent space, not the physical precision of the actual manufacturing mold. Because any inverse design framework is constrained by its underlying predictive engine, real-world deployment precision remains inevitably bound by the Virtual Metrology model’s intrinsic error (e.g., the established MAE of 0.2016 D). Consequently, while the algorithm successfully generates optimal parameters, transforming these computational prescriptions into experimentally demonstrated manufacturing accuracy requires subsequent Hardware-in-the-Loop (HIL) physical validation.

To evaluate the structural robustness of the digital twin inverse design system, automated process tuning simulations were conducted across ten distinct industrial target scenarios, spanning a wide refractive spectrum from low power (−1.00 D) to extreme myopia (−9.00 D). The optimized process parameter combinations are explicitly mapped in Table 6. The results demonstrate that the digital twin framework achieves computational convergence with maximum observed simulation errors restricted to 0.0006 D. Furthermore, the framework demonstrates a highly dynamic approach to equipment allocation. For instance, the system allocated Machine #03 (Pos #08) to satisfy the low-curvature boundary of −1.00 D but dynamically reconfigured the assignment to Machine #02 (Pos #09) when targeting −6.00 D. This fluid optimization pattern confirms that the FT-Transformer successfully internalized the unique compatibility affinities between individual equipment assets and specialized mold configurations, providing field engineers with automated, computationally optimized process prescriptions.

## 4. Discussion

### 4.1. Scientific Interpretation of Latent Embeddings

The quantitative performance enhancements must be carefully interpreted within the context of manufacturing physics and polymer thermodynamics. Within PIM systems, conventional analytical formulations often fail due to their reliance on idealized steady-state assumptions [8]. In contrast, the continuous token embedding matrices and attention maps generated by the FT-Transformer demonstrate an intrinsic capacity to absorb and characterize latent, unobservable physical variances from raw sensor data on the production floor [16]. Transforming discrete nominal identifiers, such as specific equipment numbers and multi-cavity spatial coordinates, into multi-dimensional continuous latent spaces serves as a prime example of this phenomenon. Rather than acting as arbitrary classification indices, these learnable dense vectors function as statistical proxies that quantify real-world, hardware-specific anomalies, including localized barrel heating band hysteresis, subtle nozzle clogs, and transient hydraulic clamping force decay.

The statistical consistency of the latent space is reflected by the explicit interaction weights computed between hardware units and localized cavities. In multi-machine precision operations, every individual injection molding system displays a unique thermal distribution profile and mechanical tolerance signature. Similarly, different mold cavity positions exhibit micrometer-scale geometric deviations due to non-uniform tool wear, micro-clamping misalignment, and localized cooling-line calcification [7]. When standard neural network architectures compress these variables, they systematically smooth out these critical, highly localized boundary deviations. However, the MHSA layer handles this by computing explicit cross-attention scores across heterogeneous sensor dimensions simultaneously.

The model’s prioritization of geometric design indicators—such as the inner core insert specification and outer boundaries—confirms its alignment with the principles of optical refraction physics [4]. Yet, its ability to successfully cross-reference these continuous dimensions with discrete context elements aligns closely with the hypothesis that the network successfully captured the underlying statistical associations serving as statistical indicators for equipment-mold compatibility. By digitalizing these associative distributions into auditable weights, the framework establishes a data-driven foundation for automated inverse process design.

### 4.2. Managerial and Practical Implications for Decision Support

Beyond its theoretical contributions, this framework offers immediate practical utility for the deployment of VM as a prescriptive DSS in high-stakes manufacturing environments [44]. In precision engineering sectors, the primary barrier to the integration of artificial intelligence is not a lack of predictive accuracy, but rather a profound trust deficit among domain experts and shop-floor operators [10]. When a computational surrogate operates as an opaque black box, engineers routinely reject its optimization prescriptions due to the catastrophic financial and operational risks associated with unverified autonomous adjustments. The dual-level XAI architecture proposed in this study directly resolves this institutional resistance by transforming the decision pipeline into an auditable validation mechanism. This strongly aligns with recent findings that explicit XAI integration significantly improves human–AI collaborative task performance in complex environments [45].

By exposing the internalized MHSA matrices as clear interaction topographies, the system provides field engineers with a transparent algorithmic auditing trail. Operators can visually verify whether the model’s automated parameter selections stem from statistically robust equipment-mold associative patterns—such as underlying thermodynamic affinities—or spurious data correlations. This transparency significantly enhances the human–AI relationship, shifting the operational paradigm from blind automation to structured, high-fidelity collaboration. By grounding automated prescriptive adjustments in verifiable physical constraints, the system reflects the emerging industrial paradigm of systematically quantifying the empirical robustness and reliability of attention-based explanations in high-stakes tabular deep learning deployments [42,46].

To contextualize this operational shift, consider a demanding industrial scenario involving an urgent custom manufacturing order requiring a rigid target specification of −3.75 D. In a conventional setup, operators must rely on intuition to manually configure machine parameters, run pilot batches, execute time-consuming physical metrology, and repeatedly recalibrate the hardware. This legacy methodology requires hours of machine downtime and generates substantial polymer scrap. Under the proposed digital twin framework, the inverse design optimization engine bypasses this physical loop entirely. By automatically prescribing validated machine recipes in under one second—reducing optimization latencies by orders of magnitude compared to traditional heuristic trials—the DSS drastically cuts tool setup times, minimizes raw material waste, and reduces the energy footprint of the manufacturing cell, illustrating a practical framework for agile and sustainable manufacturing [47]. While the average MAE (0.2016 D) operates below the mandatory ±0.25 D regulatory threshold, mean aggregate errors do not inherently guarantee individual sample compliance. However, as empirically validated by the 76.19% regulatory tolerance satisfaction rate derived from the residual distribution**,** the proposed VM surrogate provides a reliable numerical foundation to minimize false-positive scrap for the vast majority of continuous production cycles.

### 4.3. Methodological Limitations and Future Directions

While the proposed explainable digital twin achieves state-of-the-art predictive precision and optimization stability, a comprehensive validation requires addressing the intrinsic boundaries of offline data-driven environments.

First, regarding the absolute precision of the optimization engine, it is imperative to acknowledge the distinction between computational convergence and physical guarantees. While the GA optimization achieved an algorithmic absolute error bounded below 0.001 D, this represents an algorithmic convergence metric within the continuous latent space, not an absolute real-world physical guarantee. Such deep learning-based surrogate optimization loops are inherently susceptible to surrogate blind spots or adversarial vulnerabilities, where the algorithm may identify artificial computational optima that violate unmodeled mechanical constraints. Therefore, future studies must integrate Hardware-in-the-Loop (HIL) physical validations to verify whether these computational recipes are physically executable and precisely replicable on the actual shop floor.

Second, while the initial validation methodology relied on a randomized 5-fold CV protocol, this study explicitly executed an equipment-isolated Leave-One-Machine-Out (LOMO) validation logic (via Group K-Fold) to rigorously address the inherent risk of batch-level data leakage. Although the LOMO protocol successfully confirmed inter-machine structural generalization (yielding an $R^{2}$ of 0.8820), a primary systemic constraint remains the temporal phenomenon of concept drift, a common challenge in static industrial benchmarks [48]. Consequently, static surrogate topographies cannot spontaneously adapt to real-world environmental fluctuations, such as seasonal factory microclimate shifts or tool wear propagation. To counteract temporal concept drift without incurring prohibitive cloud-computing latencies, future work targets the integration of online continual learning mechanics directly at the manufacturing edge. This deployment paradigm will utilize Knowledge Distillation techniques to compress the dense tabular transformer infrastructure into highly lightweight student topologies [49], enabling real-time, on-device inference on microscopic edge-computing units.

Finally, extending the optimization capabilities from a single-objective GA to a multi-objective paradigm represents a vital evolutionary trajectory [50]. True production efficiency requires the simultaneous balancing of competing operational goals, including the minimization of cycle times, reduction in total hydraulic energy consumption, and mitigation of mechanical tool stress [34]. Integrating advanced multi-objective evolutionary algorithms, such as Non-dominated Sorting Genetic Algorithm II (NSGA-II), will enable the digital twin to map a multidimensional Pareto optimal front, allowing field engineers to select process recipes that achieve an optimal trade-off between established optical quality standards and macro-level sustainability metrics.

## Figures and Tables

**Table 1:** Descriptive statistics of the dataset partitioning during constraint-based preprocessing.

Stage	Sample Count	Description
Original Data	175,102	Raw industrial sensor logs acquired from the open-access KAMP benchmark repository.
Excluded	13	Missing null records (no instances violated the physical optical boundaries of −12.0 D to +6.0 D).
Final Dataset	175,089	High-fidelity data matrix utilized for model training and evolutionary optimization loops.

**Table 2:** Comprehensive hyperparameter configurations for the DNN and evolutionary optimization engine.

Component	Parameter	Value	Operational Function Inside Architecture
Neural Surrogate	Embedding Dimension (d)	64	Dimensional width of continuous latent vectors within the continuous space.
	Attention Heads (h)	8	Parallel optimization subspaces tracking orthogonal feature interactions.
	Transformer Layers	3	Block count of stacked self-attention and dense feed-forward subroutines.
	Optimization Engine	AdamW	Gradient descent optimizer with decoupled weight decay regularization.
	Learning Rate Step	1 × 10^−4^	Step size coefficient driving model parameter update trajectories.
	Batch Size Capacity	512	Single-epoch training data blocks processed simultaneously.
	Training Epochs	10	Total forward and backward passes executed per validation fold.
	Activation Routine	ReLU	Rectified Linear Unit for non-linear forward state transitions.
Evolutionary GA	Population Size (N)	50	Number of candidate equipment-recipe chromosomes evaluated per generation.
	Max Generations (G_max_)	30	Absolute iteration limit establishing complete simulation error convergence.
	Elitism Ratio	0.2	Preservation of the top 20% fittest chromosomes to prevent structural degradation.
	Mutation Rate	0.3	Probability of polynomial and categorical perturbation to maintain genetic diversity.

Note: The mutation rate ($P_{m}=0.3$) was empirically selected to prevent premature stagnation within the highly non-convex transformer latent space.

**Table 3:** Performance comparison of deep learning architectures across 5-fold CV.

Model	RMSE (D)	MAE (D)	*R^2^* Score	Inference Time (ms/Sample)
**Proposed (FT-Transformer)**	**0.4211 ± 0.0190**	**0.2016 ± 0.0066**	**0.97 ± 0.00**	**0.017**
1D-CNN (Baseline)	0.5059 ± 0.0167	0.2131 ± 0.0074	0.96 ± 0.00	0.010
ResNet (Deep MLP Baseline)	1.5617 ± 0.3271	1.1210 ± 0.2505	0.56 ± 0.20	0.013

Note: Standard deviations for the $R^{2}$ scores of FT-Transformer and 1D-CNN are present but fall below 0.01 across the 5-fold CV, reflecting highly stable convergence. Bold values indicate the best predictive performance among the evaluated models.

**Table 4:** Ablation Study on MHSA efficacy.

Variant	RMSE (D)	Relative Performance Drop	Status
Baseline DNN (No-Attention)	1.4903	−218.8% (Reference Baseline)	Non-optimal
Single-Head Attention	0.5318	−13.7%	Suboptimal
**Proposed Multi-Head (8 Heads)**	**0.4211**	**Best Performance**	**Optimal**

Note: Bold values indicate the optimal performance among the evaluated architectures.

**Table 5:** The decoded ‘Golden Recipe’ prescribing optimal equipment, mold coordinates, and geometric boundaries for a target specification of −3.25 D.

Category	Variable	Optimal Value	Description
Equipment	EQUIP_ID	Machine #05	Selected optimal manufacturing unit.
Mold Setup	MOLD_POS	Position #12	Localized multi-cavity coordinate.
	MOLD_IN_TOP	Core-Type 11	Inner top core insert specification.
	MOLD_IN_BOT	Core-Type 04	Inner bottom core insert specification.
	MOLD_OUT_TOP	Core-Type 07	Outer top core insert specification.
	MOLD_OUT_BOT	Core-Type 07	Outer bottom core insert specification.
Process Param	IN_RADIUS	7.7220 mm	Derived optimal inner geometric boundary.
	OUT_RADIUS	8.1585 mm	Derived optimal outer geometric boundary.
Result	Target/Pred	−3.2500 D/−3.2499 D	Absolute Simulation Error: 0.0001 D

**Table 6:** Full optimization recipes and algorithmic absolute simulation errors across ten target scenarios.

Target (D)	Optimal Equipment	Optimal Position	Inner Radius (mm)	Outer Radius (mm)	AI Prediction (D)	Abs Error (D)	Status
−1.00	Machine #03	Pos #08	7.8498	8.4852	−1.0006	0.0006	Pass
−2.00	Machine #08	Pos #05	7.6624	8.1174	−1.9994	0.0006	Pass
−3.00	Machine #04	Pos #06	7.5082	8.2910	−2.9999	0.0001	Pass
−3.25	Machine #05	Pos #12	7.7220	8.1585	−3.2499	0.0001	Pass
−4.00	Machine #04	Pos #03	7.5599	7.9140	−3.9996	0.0004	Pass
−5.00	Machine #07	Pos #05	7.4867	8.0580	−5.0005	0.0005	Pass
−6.00	Machine #02	Pos #09	7.5769	7.8690	−6.0001	0.0001	Pass
−7.00	Machine #04	Pos #08	7.1964	7.8050	−6.9999	0.0001	Pass
−8.00	Machine #02	Pos #06	7.1035	7.6988	−7.9997	0.0003	Pass
−9.00	Machine #04	Pos #02	6.9125	7.6527	−8.9996	0.0004	Pass

## Data Availability

The raw manufacturing sensor datasets analyzed during the current study were derived from the open-access repository hosted by the Korea AI Manufacturing Platform (KAMP), officially managed by the Ministry of SMEs and Startups (MSS) and KAIST (available at https://www.kamp-ai.kr).

## Abbreviations

The following abbreviations are used in this manuscript:

1D-CNN	1-Dimensional Convolutional Neural Network
CV	Cross-Validation
D	Diopters
DNN	Deep Neural Network
DSS	Decision Support System
FFN	Feed-Forward Network
FT-Transformer	Feature Tokenizer Transformer
GA	Genetic Algorithm
GPU	Graphics Processing Unit
HIL	Hardware-in-the-Loop
IoT	Internet of Things
IQR	Interquartile Range
KAMP	Korea AI Manufacturing Platform
KDE	Kernel Density Estimation
KIML	Knowledge-Informed Machine Learning
LOMO	Leave-One-Machine-Out
MAE	Mean Absolute Error
MES	Manufacturing Execution System
MHSA	Multi-Head Self-Attention
MLP	Multi-Layer Perceptron
MSS	Ministry of SMEs and Startups
NSGA-II	Non-dominated Sorting Genetic Algorithm II
PFI	Permutation Feature Importance
PIM	Precision Injection Molding
PLC	Programmable Logic Controller
R^2^	Coefficient of Determination
ResNet	Deep Residual Network
RMSE	Root Mean Squared Error
SBX	Simulated Binary Crossover
VM	Virtual Metrology
VRAM	Video Random Access Memory
XAI	Explainable Artificial Intelligence
ZDM	Zero-Defect Manufacturing
